# Does an adapted Dialectical Behaviour Therapy skills training programme result in positive outcomes for participants with a dual diagnosis? A mixed methods study

**DOI:** 10.1186/s13722-019-0156-2

**Published:** 2019-08-15

**Authors:** Daniel Flynn, Mary Joyce, Ailbhe Spillane, Conal Wrigley, Paul Corcoran, Aoife Hayes, Marian Flynn, David Wyse, Barry Corkery, Brid Mooney

**Affiliations:** 1 Cork Mental Health Service, Cork Kerry Community Healthcare, Health Service Executive, St Finbarr’s Hospital, Cork, Ireland; 2 National Suicide Research Foundation, University College Cork, Western Gateway Building, Western Road, Cork, Ireland; 3 Cork Kerry Community Healthcare Addiction Service, Arbour House, St Finbarr’s Hospital, Douglas Rd., Cork, Ireland

**Keywords:** Dialectical Behaviour Therapy, Effectiveness, Dual diagnosis, Adults, Borderline Personality Disorder, Emotionally unstable personality disorder, Substance misuse

## Abstract

**Background:**

Treating severe emotional dysregulation and co-occurring substance misuse is challenging. Dialectical behaviour therapy (DBT) is a comprehensive and evidence-based treatment for borderline personality disorder (BPD). It has been hypothesised that the skills training, which is a facet of the full DBT programme, might be effective for people with severe emotional dysregulation and other co-occurring conditions, but who do not meet the criteria for BPD. However, there is limited research on standalone DBT skills training for people with substance misuse and emotional dysregulation.

**Methods:**

A mixed methods study employing an explanatory sequential design was conducted where participants with a dual diagnosis (n = 64) were recruited from a community-based public addiction treatment service in Ireland between March 2015 and January 2018. DBT therapists screened potential participants against the study eligibility criteria. Quantitative self-report measures examining emotion regulation, mindfulness, adaptive and maladaptive coping responses including substance misuse, and qualitative feedback from participants were collected. Quantitative data were summarised by their mean and standard deviation and multilevel linear mixed effects models were used to estimate the mean change from baseline to post-intervention and the 6-month follow-up period. Thematic analysis was used to analyse the qualitative data.

**Results:**

Quantitative results indicated reductions in binge drinking and use of Class A, B and C drug use from pre-intervention (T1) to the 6-month follow-up (T3). Additionally, significant improvements were noted for mindfulness practice and DBT skills use from T1 to T3 (p < 0.001). There were also significant reductions in dysfunctional coping and emotional dysregulation from T1 to T3 (p < 0.001). Significant differences were identified from pre to post intervention in reported substance use, *p *= 0.002. However, there were no significant differences between pre-intervention and 6-month follow up reports of substance use or at post-intervention to 6 month follow up. Qualitative findings indicated three superordinate themes in relation to participants’ experiences of a DBT skills training programme, adapted from standard DBT: (1) new lease of life; (2) need for continued formal aftercare and (3) programme improvements. Participants described reductions in substance misuse, while having increased confidence to use the DBT skills they had learned in the programme to deal with difficult emotions and life stressors.

**Conclusions:**

This DBT skills training programme, adapted from standard DBT, showed positive results for participants and appears effective in treating people with co-occurring disorders. Qualitative results of this mixed methods study corroborate the quantitative results indicating that the experiences of participants have been positive. The study indicates that a DBT skills programme may provide a useful therapeutic approach to managing co-occurring symptoms.

## Background

Dialectical behaviour therapy (DBT) is a well-established comprehensive evidence-based treatment that was primarily developed for individuals with borderline personality disorder (BPD) and for those who are chronically suicidal [1, 2]. BPD is characterised by severe disturbances in emotional regulation [3]. An estimated 1% of the population meet the criteria for BPD [4]. However, given the diagnostic complexities of emotional dysregulation in clients with co-occurring presentations, identifying accurate rates within community mental health settings can often be challenging [5].

Prevalence rates of BPD in community mental health settings ranges from 10 to 30% in outpatient and inpatient settings [6]. When evaluated within distinctive groups of disorders, dual diagnoses rates of BPD and substance use disorders can rise to 65% [7]. Moreover, while much is known on the effectiveness of transdiagnostic approaches to treatment for BPD and substance use disorders, more research is needed to evaluate the effectiveness of treatment approaches which are specifically tailored to target co-occurring presentations of personality disorder traits and substance misuse.

To date, two systematic reviews have investigated effective treatments options for co-occurring BPD and substance use disorders [8, 9]. The most recent review, conducted in 2015, identified a total of ten studies which included four DBT interventions [8]. These studies identify the potential for DBT to be effective for co-occurring disorders. For example, a study carried out by Linehan and colleagues found a significant reduction in substance use disorder (SUD) symptoms for participants in DBT [10, 11]. However, it was difficult to evaluate the contribution of DBT alone given the inclusion of medication in the study [11]. Harned and colleagues compared 101 women receiving DBT (*n* = 52) and those receiving Community Treatment by Experts (CTBE, *n *= 49) [12]. The DBT group were significantly more likely to achieve full remission from substance use disorders than the CTBE patients and spent more time in partial remission than the comparison group. A further study comparing the effectiveness of DBT to comprehension validation therapy for people receiving treatment for opiate addiction showed a significant reduction in opiate use for the DBT group [10]. However, no significant differences were found between the DBT and treatment as usual group when investigating the effectiveness of DBT on SUDs with non-specific personality disorders [13]. Recently, in 2018, a pilot randomised controlled trial (RCT) examined the efficacy of internet-delivered DBT skills training for suicidal people engaging in heavy episodic alcohol consumption [14]. Individuals who received the intervention (n = 30) showed faster reductions in alcohol consumption compared to the waitlist controls (n = 30). There were significant reductions in all outcome measures including emotional dysregulation and severity of alcohol use during the 16-week study period [14].

Although these studies have identified the effectiveness of DBT in co-occurring disorders, further studies are required to extend the existing literature in evaluating the effectiveness of adapted DBT programmes [12]. Group skills training is one of the four modes of DBT treatment [15] and in recent years has become a standalone treatment to help patients engaging in a range of maladaptive behaviours including substance misuse [16]. Skills only training aims to increase goal-oriented behaviours, which focuses on four skills modules: mindfulness, emotional regulation, interpersonal effectiveness and distress tolerance [2, 15]. A recent open trial showed an improvement in alcohol-related behaviour for alcohol dependent individuals who received a 3 month DBT skills training programme [2]. However, overall there is limited research on standalone DBT skills training for people with substance misuse.

While the majority of studies in this area are highly controlled clinical trials, there is a paucity of real-world studies of DBT in clinical practice, with some notable exceptions [17]. A study published 20 years ago indicated that just 9% of people with alcohol dependence who attempted suicide were receiving psychotherapy in the month after the index attempt. This research highlights the unmet treatment needs of this client group within clinical practice. Therefore, the introduction of DBT skills training for this client group may address this unmet need phenomenon in clinical practice. The intervention described in this study was borne out of a need to develop an effective treatment for people with a dual diagnosis in a real-world public health system with associated resource demands and waiting time periods. The present study therefore represents a real-world view of DBT skills training as an intervention in a naturalistic setting within clinical practice. Given the paucity of research in this area, this study aims to address this gap in the literature by evaluating the effectiveness of a 24-week DBT skills-only programme for individuals who present with both emotional dysregulation and substance misuse.

## Method

### Design and study setting

The study applied a mixed methods approach, using an explanatory sequential design where quantitative data collection was followed by qualitative data collection. The quantitative component of the study examined self-reported measures on the effectiveness of the Understanding and Managing Emotions-Addiction (U&ME-A) programme, which is a targeted 24-week group skills programme derived from DBT. These quantitative measures were collected in the form of survey data which was also supplemented with self-reported qualitative feedback from participants. Therefore, the qualitative sample was a subset of the larger quantitative sample. This study was carried out in Arbour House, between March 2015 and January 2018 in Ireland. Arbour House is a community-based public health addiction treatment service for people with alcohol and substance misuse, as well as other addiction issues. Senior addiction counsellors and DBT therapists facilitated the introduction of and collected data for the U&ME-A programme.

### Sample and recruitment

Participants were individuals with a dual diagnosis (n = 64) who were attending a public community-based adult addiction service with mental health and addiction diagnoses. The diagnosis of SUD was made by a detailed structured interview following the World Health Organisation (WHO) classification of alcohol use (hazardous, harmful or dependent) and the WHO definition of high or low-risk alcohol consumption. Participants were excluded if they had one or more incidents of self-harm within the past 6 months or if severe developmental delays, cognitive impairment or learning difficulties were present. Participants were also excluded if they had an active psychosis or a primary diagnosis for which other treatments were recommended. All individuals who participated in U&Me-A were invited to participate in the research study. The DBT therapist [BC, BM] informed each suitable service user about the study. At baseline, a research team member or DBT therapist briefed the service users, obtained written informed consent and administered the measures. DBT therapists delivering the programme undertook data collection at all other time points.

### Treatment

The treatment provided in the programme was an adapted version of the group skills training component of standard DBT [15]. In standard DBT, the group skills curriculum comprises three modules which are delivered once a week by multidisciplinary DBT therapists over 24 weeks [18]. These modules are then repeated, extending the programme duration of 48 weeks. This DBT skills training programme followed the same format as standard DBT group skills with the exception that the three modules were delivered once and not repeated, resulting in a shorter 24-week programme. Initially each module comprised 8 weekly sessions [18]. However, 7 months into the programme and following consultation with the DBT programme developer (Prof. Marsha Linehan), the emotional regulation module was extended to 9 weeks and the interpersonal effectiveness module was reduced to 7 weeks (Table 1). Consequently, the programme duration remained at 24 weeks. Each group session was structured and delivered by two DBT therapists [15]. Each participant in the programme was also linked with a key worker from the U&Me-A team. Participants met with their key worker on a monthly basis to review progress and obtain clarity on elements of the programme as necessary.

**Table 1:** Module content of the U&ME—a programme

Module	Content
1	Two mindfulness skills training workshops and six distress tolerance skills training workshops
2	Two mindfulness skills training workshops and seven emotional regulation skills training workshops
3	Two mindfulness skills training workshops and five interpersonal effectiveness skills training workshops

### Measures

Four self-report measures were administered at each time-point for this study. The Difficulties in Emotional Regulation Scale (DERS) is a 36-item scale that was used to measure the construct of emotional regulation, with an internal reliability of 0.93 [19]. The Five Facet Mindfulness Questionnaire (FFMQ) is a 24-item scale that measures the construct of mindfulness, with internal reliability ranging from 0.73 to 0.91 on the different facets of the scale [20]. The Dialectical Behaviour Therapy Ways of Coping Checklist (DBT-WCCL) is a 59-item scale that measures participants’ adaptive (the DBT skills use subscale; 38 items) and maladaptive coping responses (the dysfunctional coping subscale; 21 items) to difficult situations over the past month [21]. The internal reliability of the scale is 0.92 to 0.96. Finally, the Cork Impact of Substance Misuse Scale (CISMS), which was developed specifically for this study, gathered information about frequency and severity of substance misuse, and measures emotions and feelings related to substance misuse and participants' perceived recovery on a Likert type scale. Cronbach's alpha for the emotions and feelings subscales were 0.96, and 0.70 for the recovery scale.

A mixed methods questionnaire was also developed for the purpose of this study and was administered following completion of the intervention and at follow-up. The quantitative component of the questionnaire asked participants to rate their experiences of the programme on items such as how useful they found the material and how often they used the skills they learned. The qualitative component of this questionnaire asked participants to describe their experiences of the intervention. There were three time-points for data collection: quantitative measures were collected at baseline (T1—start of intervention); 6 months (T2—end of intervention); and 12 months (T3—6 months post-intervention). The mixed method questionnaire was administered at T2 and T3 only.

### Analysis

All quantitative self-report outcome measures were summarised by their mean and standard deviation. For these measures, multilevel linear mixed-effects regression models were used to estimate the mean at baseline (T1) and the mean change from baseline to each follow-up (T2 and T3). Mixed-effects models use all available data at each time-point rather than the data from individuals assessed at all times. Based on the results of likelihood ratio tests, we included in all models a random intercept to adjust for random heterogeneity between subjects in the baseline level of the outcome measure. We also included a random slope to adjust for random heterogeneity of between subjects in change over time and allowed unstructured covariance between these random effects. Descriptive statistics and non-parametric statistics were used to examine reductions in substance use over time. Data were analysed using Stata version 13.1 and IBM SPSS Statistics 22.0 for Windows. Thematic analysis was used to analyse the qualitative data. Thematic analysis is not a theoretically bounded method but one which allows for a variety of ontological and epistemological stances [22]. Thematic analysis consists of a number of discreet steps, including familiarisation with the data, generating initial codes, searching, reviewing and finally, defining overarching themes [22]. Two authors that were not involved in the data collection or administering the programme [AS, CW] coded the data.

## Results

### Quantitative

Sixty-four participants provided written informed consent to participate in this study. Data were available for 63 participants (n = 24 males, n = 39 females) at baseline (see Table 2). Seventeen participants (26.5%) had traits of BPD or traits of emotionally unstable personality disorder (EUPD). Other co-occurring morbidities in study participants included depression, bipolar, anxiety and mood disorders. The majority of participants were aged between 35 and 44 years, single and unemployed. Nearly three-quarters of participants (n = 47, 73%) had previously engaged in treatment for addiction either in the public or private system, while just over half (n = 34, 53%) were currently engaged in a care plan with the service prior to their referral to the programme. Sixty-seven percent (n = 43) of participants had a history of suicidal ideation, attempted suicide or self-harm. Due to the volume of missing data at post-intervention, Little’s Chi square test was conducted to evaluate if values were missing completely at random (MCAR). Categorical variables (gender, relationship status, employment status) were crosstabulated with continuous data (age, DBT skills). Little’s test revealed the data was missing at random, *X*^2^ (*DF* = 27) = 16.99, *p* = 0.93.

**Table 2:** Baseline characteristics of study sample

Characteristics	N (%)
Sex (n = 63)	
Male	24 (38.1)
Female	39 (61.9)
Age (n = 62)	
18–34 years	19 (30.6)
35–44 years	24 (38.7)
45+ years	19 (30.6)
Marital status (n = 63)	
Single	27 (42.9)
In a relationship	16 (25.4)
Married	10 (15.9)
Separated/divorced	10 (15.9)
Employment status (n = 62)	
Full/part-time	14 (22.6)
Unemployed	21 (33.9)
Retired/other/homemaker	11 (17.7)
Student	8 (12.9)
Disability	8 (12.9)

### Referral data

Referral data indicated that at baseline, the majority of participants (61%) were seeking treatment for misuse/dependence of a single substance (Inc. alcohol, amphetamines benzodiazepines cannabis, cocaine codeine and heroin). Just over one quarter (26.5%) were seeking treatment for misuse/dependence of two substances. One in ten (12.5%) were seeking treatment for misuse/dependence of three or more substances. Alcohol was the most commonly reported substance (73%) for which participants were seeking treatment, followed by sedatives/stimulants (25%), cannabis (25%) and opioids (17%).

### Self-reported substance use (CISMS)

Participants were asked to record any intake of substances over the last month at pre-intervention, post-intervention and follow up. At pre-intervention, 63% indicated they had used substances in the month prior to treatment, while 37% said they had not (*n* = 62). For those who completed the programme, 19% reported substance use in the previous month and 81% reported no substance use. For those who provided follow-up data (n = 19), 37% indicated they had used substances and 63% reported no use. Cochran’s Q test was used to examine significant differences between pre, post and follow up reported substance use. Statistically significant differences were found over time for reported substance use, *X*^2^(2) = 9.33, *p *= 0.01. To identify if these differences were significant across all three time-points, a series of post hoc analyses were conducted using an exact Mcnemar’s test with a Bonferroni correction of *p *< 0.017. Significant differences were identified from pre to post intervention in reported substance use, *p *= 0.002. However, there were no significant differences between pre-intervention and 6-month follow up reports of substance use (*p *= 0.25) or at post-intervention to 6 month follow up (*p *= 0.06).

Table 3 reports changes in substance use from baseline (T1), post-intervention (T2) to 6 months follow-up (T3), as measured by the CISMS. Individuals who reported engaging in excessive (binge) drinking reduced from 14 at T1 to four at T3. Drug consumption was separated into three categories; Class A (the most harmful), Class B (an intermediate category) and Class C (less harmful). Four individuals reported use of Class A drugs (including cocaine, heroin, and ecstasy) at T1, which reduced to one person at T3. Similarly, self-reported Class B drug consumption (including barbiturates, cannabis and codeine) reduced from 12 at T1 to three at T3. No participant reported Class C drug consumption (inc. benzodiazepines and anabolic steroids) at T3, compared to seven reporting use at baseline (T1).

**Table 3:** Alcohol and substance use at each time-point

Self-report substance use in past 30 days	Yes (*n*)	No (*n*)	Missing/not indicated (*n*)
Substance use at baseline (T1)			
Alcohol ≥ 6 Units^a^	14	10	32
Alcohol ≤ 6 Units^b^	8		
Class A drugs	4	41	19
Class B drugs	12	34	18
Class C drugs	7	35	22
Substance use at post-intervention (T2)			
Alcohol ≥ 6 Units^a^	2	20	35
Alcohol ≤ 6 Units^b^	7		
Class A drugs	2	23	39
Class B drugs	4	24	36
Class C drugs	2	19	43
Substance use at follow-up (T3)			
Alcohol ≥ 6 Units^a^	4	16	44
Alcohol ≤ 6 Units^b^	3		
Class A drugs	1	12	51
Class B drugs	3	11	50
Class C drugs	0	6	58

^a^Indicates excessive (binge) drinking levels

^b^Indicates below excessive drinking levels

Multilevel linear mixed-effects regression models were used to estimate the mean at T1 and the mean change from T1 to T2 and T1 to T3. Significant pre-post differences were found for dysfunctional coping, DBT skills use, emotional dysregulation and mindfulness, and also from pre-intervention to follow-up (Table 4). There were no significant differences in scores from T2 to T3.

**Table 4:** Outcome measure estimated baseline means (M) and changes at subsequent time-points

Variable	Estimate T1 M (95% CI)	Change at T2 M (95% CI)	Change at T3 M (95% CI)
Dysfunctional coping (DBT WCCL)	43.82 (41.41, 46.23)	− 11.63* (− 15.54, − 7.71)	− 13.60* (− 18.77, − 8.42)
DBT skill use (DBT WCCL)	65.00 (60.19, 69.81)	+ 23.64* (18.48, 28.80)	+ 20.58* (13.66, 27.50)
Emotional dysregulation (DERS)	120.97 (115.68, 126.27)	− 41.64* (− 50.34, − 32.94)	− 38.97* (− 50.75, − 27.18)
Mindfulness (FFMQ)	64.48 (61.88, 67.07)	+ 18.05* (13.30, 22.81)	+ 18.52* (12.03, 25.01)

*Note* Changes at each time-point are relative to the baseline

*95% CI* 95% confidence interval

* *p *< .001

### Mixed methods evaluation

For the quantitative element of the questionnaire which explored participant's experience of the programme, at 6 months post-intervention (T3), the majority of participants (92%) reported that the material covered in the programme was ‘very much useful to them’, with the remaining 8% of participants reporting that it was ‘somewhat useful’. In the six months after completing the programme, three in four participants (75%) reported they used the skills ‘very often’; the remaining quarter (25%) reported using the skills ‘often’. The majority of participants (87.5%) reported that completing the programme has helped them to deal more effectively with difficulties experienced, while the remainder (12.5%) felt that it helped them ‘somewhat’ to deal with difficulties.

Three main themes were identified from the qualitative analysis process: ‘*new lease of life*’, ‘*need for continued formal aftercare*’ and ‘*programme improvements*’.

#### New lease of life

This first superordinate theme has three subthemes: ‘*reduced substance misuse and behaviour dysregulation*’, ‘*increased confidence/assertiveness*’ and ‘*new insights into self and addiction*’. It was apparent from written descriptions of participants’ experiences of the programme at the end of the intervention (T2) and at the 6 month follow-up (T3) that the programme sparked a significant improvement both in reduced substance misuse and also overall benefits to their perspective and outlook on life. Participants spoke about how they “*truly feel that [the programme] has “saved my life”*’ and will be “*forever grateful for the life you [the programme] have given me”*. A number of participants felt “*privileged to have been part of it”*, where they felt their lives were “*only beginning now”*. Participants described how skills learned during the programme “*have very much replaced the drink in dealing with my emotions”*, helped them to “*stay clean”* and that they no longer rely on “*alcohol as a crutch”* in dealing with life’s challenges. Some participants spoke specifically about the difficulties they found themselves in as a direct result of their substance misuse and were grateful that they had broken this cycle:

“[*I am] no longer going on drink binges for days on end. I haven’t been admitted to hospital for a while or gone to my doctor for alcohol abuse. [I am] extremely happy with the programme*”

Others described how they would drink alcohol “*to alleviate or blot out*” mental health problems, including depression and anxiety, but now use the skills learned in the programme to manage their emotions. Improvements in behaviour were noted by many participants as they no longer “*fly off the handle when a stressful situation arises”.* A number of participants spoke about how the programme has not only changed their own lives but also their family member’s lives for the better:

“*I’m sober two years this month and the greatest gift I got was to be so lucky to have the chance to do the [name of programme], it has changed my life for the better…I am so, so grateful for this and to pass the skills on to my daughters by the way I now behave, I have also seen a change in my daughters because of this programme. Thank you so much, I really and truly feel that you have saved my life”*

Increased confidence was often noted by participants in “*dealing with everyday life*” and “*dealing with people*”. Others felt the skills learned during the programme gave them the confidence and “*freedom to be me*”. Furthermore, the programme gave participants the confidence and skills to deal effectively with challenges encountered in life, as opposed to becoming overwhelmed by difficult thoughts and emotions:

*“[I am] less likely to be overwhelmed by events, life will continue to bring new demands…but I do not fear and let them destroy me as they nearly did in the past…I am more assertive in the way I control my life, making decisions no matter how difficult they may seem to be…”*

Participants often described gaining new insights about themselves and about their addiction through the programme. For some, they learnt that alcohol is a depressant “*so it should and must be a no*-*go area for me*”. Others learned to be more self-compassionate as they “*face the daily struggles of life*”. It also became clear to some why they engaged in substance misuse and other emotionally dysregulated behaviours:

“*At long last I understand why I had been behaving [the way I was] and generations of habit I have now broken”*

The programme allowed participants the opportunity to reflect on their past experiences and personal life events that influenced their addictions and dysregulated behaviour.

#### Need for continued formal aftercare

This second superordinate theme has one subtheme: ‘*maintaining gains achieved’*. Continued formal support was deemed valuable by participants in order to maintain their use of the skills learned in the programme. Specifically, several participants spoke of wanting the programme to have been longer to optimise gains achieved:

“*It would be very beneficial and help us to keep in touch with skills because they are life changing skills and I can’t for the life of me figure out why such a short period of time was given to us to complete the course and learn such vital skills which will ultimately lead to a lot more people living clean and happy and much more fuller lives*”

A number of participants felt aftercare would have kept them “*on the right track for myself and my mental health*”, as they conceded that “*there’s nothing bullet proof*” and “*even the best of people make mistakes*”. One particular participant felt the lack of a structured aftercare programme may increase the risk of some individuals reverting back to their addictive behaviour in the future:

“*It’s very important that there is some sort of aftercare programme with this. Support is needed after. I imagine a lot of people are failing because of this*”

#### Programme improvements

Most participants felt that no element of the programme needed to be changed, as they described it as a “*brilliant course*” and “*an invaluable experience*” which has “*definitely changed my life for the better*”. Participants were especially grateful for the “*kindness, respect and compassion*” shown by the programme facilitators:

“*The counsellors deserve great praise in their attitude towards people with addictions. There is never any putdown or negative criticism* - *only positivity and validation if you are showing effort and cooperation*”

Notwithstanding this, a number of participants wanted the programme to be longer, especially as “*extra time*” was required “*to avoid rushing home tasks*”. Lack of sufficient time may have also impacted on a small number of participants being unable to effectively complete tasks at home:

“*I found it hard to understand when we were advised to complete it at home*”

One participant noted that “*more family involvement during treatment*” would have been beneficial. Participants also suggested that this programme be rolled out in a “*child*-*friendly way*”, in terms of content and age-appropriateness, to equip school-aged children with the tools and skills required to live a “*healthy life*”. Extending the programme to children and young people was deemed appropriate given “*this era of poor mental health and addictions in young people*”.

## Discussion

The findings of this study show significant improvements in emotional regulation, mindfulness, DBT skills use and dysfunctional coping for participants who completed the U&Me-A programme. Additionally, there were noted reductions in self-reported substance misuse from pre to post-intervention and pre to the 6-month follow-up. Results from the qualitative component of this study suggest that the DBT skills training programme helped participants to become more confident and self-assured as they navigated the recovery process.

When the quantitative and qualitative aspects of this study are combined, they produce a more nuanced understanding of effective treatment and recovery for those with a dual diagnosis. While the qualitative findings corroborate the positive changes noted in the quantitative results, particularly in relation to reductions in or abstinence from substance use, the qualitative component of the study provides additional insights on improvements in confidence, self-assurance and the need for continued formal aftercare. In this way, the qualitative data complements the quantitative data as it provides a clearer picture of the improvements to participants’ way of living, outlook on life and coping with stressful situations, which may not have been best measured through quantitative methods. In addition to corroborating with the quantitative findings, the qualitative results also add further insights into the support needs of people with a dual diagnosis. Participants emphasised that while 24 weeks of DBT skills training helped them with reduction/abstaining from substance misuse and emotional and behavioural regulation, they felt they were not infallible and needed formal support in the long-term to maintain these gains. Given the high degree of quantitative missing data in relation to substance misuse, the qualitative results accounted for this through rich participant descriptions. Participants described how the skills training aided them in learning new mechanisms to cope with stress that previously would have involved engaging in substance misuse. The present findings are consistent with previous studies investigating the effectiveness of treatment of co-occurring substance use disorders and BPD [10–12]. There is a growing body of evidence to support the benefits of DBT skills training in reducing difficulties in emotional regulation, maintaining interpersonal relationships and increasing distress tolerance in populations with BPD who have a co-occurring substance dependency. Neacsiu and colleagues found that participants treated with a DBT skills use intervention reported three times more skills at the end of treatment than those who received treatment-as-usual [23]. The researchers found a significant reduction in suicide attempts and reported depression indicating the increased skills use could be a mechanism for changing suicidal thoughts and behaviours. The current findings indicate that skills use was significantly improved and emotional dysregulation and emotional distress significantly reduced at the end of treatment in participants with a dual diagnosis. Mindfulness meditation skills have been consistently found to reduce substance misuse as a stand-alone treatment [24, 25]. Patients with BPD and SUD often experience increases in impulsivity. Thinking patterns can be conflicted, leading to poor decision making and relapse. Mindfulness skills teach patients to look at their previous experiences and become aware of the decisions they make, leading to reductions in impulsivity. The current study found improvements in mindfulness skills which were maintained at follow-up for the study participants. Our results support the growing body of evidence for the effectiveness of DBT skills on treating these challenging presentations. Providing clients with the tools and knowledge base to effectively identify, accept and change their behaviours allows them to take ownership of their own abilities and manage their own emotions outside and beyond treatment clinics.

### Strengths and limitations

This study has a number of strengths including the mixed methods approach utilised. Mixed methods studies combine the advantages of quantitative and qualitative studies, while also bridging the gap between the inherent limitations of both approaches [26]. Bringing together quantitative and qualitative methods adds value to the research, where one gains a better understanding of the problem compared to using either method in isolation [26]. This study was also the first of its kind to be conducted in Ireland and was carried out in a publicly funded health system, the Health Service Executive (HSE). This study has demonstrated that the U&ME-A programme may be effective in treating the complex needs of people with co-occurring addiction and mental health diagnosis (including BPD/EUPD traits) and can be resourced effectively in a real-world community setting.

This study has some limitations that require consideration. The primary limitation of this research is the lack of a control group. The absence of a control group makes it difficult to determine if the observed changes in the outcome measures were a result of the intervention rather than other factors. However, given that programme participants had mental health diagnoses, addictions and severe emotional dysregulation at baseline, the findings provide a promising avenue for the treatment of dual diagnosis which historically has been predominantly viewed as challenging to treat. Another limitation is that substance use was self-reported by participants, which may be subject to social desirability bias. The quantitative questionnaire used to collect information on substance misuse will require modification for future research as it asked participants to state the number of days in the past month that they had engaged in substance use. Due to the ambiguity of this question, participants may have left this section blank if they had not engaged in substance use. This resulted in the researchers being unable to definitively say that these participants had not engaged in substance misuse. The design of the measure did not best allow for the collection of data from participants that reported no substance use in the past month. Consequently, the number of people reporting a reduction in substance misuse in this study is likely to be an underestimation of the true value. Finally, the effect of prescribed medications on outcomes for the U&Me-A treatment cohort is unknown, as we did not include measures for same in our study.

### Future research

Future community-based controlled studies are required to confirm whether the positive changes reported in this study are because of the intervention, particularly with the use of a treatment-as-usual control group. Control group studies might consider measuring treatment outcomes for other dual diagnosis interventions for service users such as individual addiction counselling, other multidisciplinary treatments, or other group treatment programmes. More structured and effective communications were developed between addiction and mental health disciplines which was a secondary benefit of undertaking this research study. Further qualitative research might explore the factors which facilitated this improvement in effective collaborative structures, especially given the knock-on benefits to service users with a dual diagnosis. Observations of the DBT skills training team suggest that future studies might examine changes in visits to emergency departments and inpatient admissions over time. This current study did not have ethical approval to examine data collected in diary cards or urinalysis/breath analysis, which is gathered routinely for clinical purposes over the course of treatment in the addiction service. Future studies would benefit from examining these corroborating data. Additionally, it would also be important that future research collects more specific socio-demographic information, mental health and substance misuse diagnoses in order to control for same in the analyses.

## Conclusions

The findings of this study indicate that the use of a standalone DBT skills training programme may result in positive outcomes for participants and appears effective in treating people with co-occurring disorders. Self-reported substance misuse decreased, while there were increases in skills adaptation which were maintained throughout the treatment period. Qualitative results of this mixed methods study corroborate the quantitative results indicating that the experiences of participants in this programme had been largely positive. The current study indicates that a standalone DBT skills programme may provide a useful therapeutic approach to managing co-occurring symptoms and provide service users with effective coping skills that are maintained over time.

## Data Availability

The datasets generated and analysed during the current study are not publicly available due to the relatively small number of participants and the specific geographic location, it would not be appropriate to publicly share this data due to the risk of people being potentially identified.

## Abbreviations

DBT: Dialectical Behaviour Therapy
BPD: Borderline Personality Disorder
CTBE: Community Treatment by Experts
CISMS: Cork Impact of Substance Misuse Scale
DERS: Difficulties in Emotional Regulation
FFMQ: Five Facet Mindfulness Questionnaire
DBT-WCCL: Dialectical Behaviour Therapy-Ways of Coping Checklist

## References

[1] Dimeff LA, Linehan MM (2008). Dialectical behavior therapy for substance abusers. Addict Sci Clin Pract..

[2] Maffei C, Cavicchioli M, Movalli M, Cavallaro R, Fossati A (2018). Dialectical behavior therapy skills training in alcohol dependence treatment: findings based on an open trial. Subst Use Misuse.

[3] McMain S, Korman LM, Dimeff L (2001). Dialectical behavior therapy and the treatment of emotion dysregulation. J Clin Psychol.

[4] Lenzenweger MF, Lane MC, Loranger AW, Kessler RC (2007). DSM-IV personality disorders in the National Comorbidity Survey Replication. Biol Psychiatry.

[5] Carpenter RW, Trull TJ (2013). Components of emotion dysregulation in borderline personality disorder: a review. Curr Psychiatry Rep..

[6] Trull T, Jahng S, Tomko RL, Wood PK, Sher KJ (2010). Revised NESARC personality disorder diagnoses: gender, prevalence, and comorbidity with substance dependence disorders. J Pers Disord.

[7] Trull T, Sher KJ, Minks-Brown C, Durbin J, Burr R (2000). Borderline personality disorder and substance use disorders: a review and integration. Clin Psychol Rev..

[8] Lee NK, Cameron J, Jenner L (2015). A systematic review of interventions for co-occurring substance use and borderline personality disorders. Drug Alcohol Rev.

[9] Pennay A, Cameron J, Reichert T, Strickland H, Lee NK, Hall K (2011). A systematic review of interventions for co-occurring substance use disorder and borderline personality disorder. J Subst Abuse Treat.

[10] Linehan M, Dimeff LA, Reynolds SK, Comtois KA, Welch SS, Heagerty P (2002). Dialectical behavior therapy versus comprehensive validation therapy plus 12-step for the treatment of opioid dependent women meeting criteria for borderline personality disorder. Drug Alcohol Depend.

[11] Linehan M, Schmidt H, Dimeff LA, Craft JC, Kanter J, Comtois KA (1999). Dialectical behavior therapy for patients with borderline personality disorder and drug-dependence. Am J Addict..

[12] Harned MS, Chapman AL, Dexter-Mazza ET, Murray A, Comtois KA, Linehan MM (2008). Treating co-occurring Axis I disorders in recurrently suicidal women with borderline personality disorder: a 2-year randomized trial of dialectical behavior therapy versus community treatment by experts. J Consult Clin Psychol.

[13] van den Bosch LMC, Verheul R, Schippers GM, van den Brink W (2002). Dialectical Behavior Therapy of borderline patients with and without substance use problems: implementation and long-term effects. Addict Behav.

[14] Wilks CR, Lungu A, Ang SY, Matsumiya B, Yin Q, Linehan MM (2018). A randomized controlled trial of an Internet delivered dialectical behavior therapy skills training for suicidal and heavy episodic drinkers. J Affect Disord.

[15] Linehan M (1993). Cognitive-behavioral treatment of borderline personality disorder. Diagnosis and treatment of mental disorders.

[16] Valentine SE, Bankoff SM, Poulin RM, Reidler EB, Pantalone DW (2015). The use of dialectical behavior therapy skills training as stand-alone treatment: a systematic review of the treatment outcome literature. J Clin Psychol.

[17] Suominen KH, Isometsä ET, Henriksson MM, Ostamo AI, Lönnqvist JK (1999). Treatment received by alcohol-dependent suicide attempters. Acta Psychiatr Scand.

[18] Flynn D, Kells M, Joyce M, Corcoran P, Gillespie C, Suarez C (2017). Standard 12 month dialectical behaviour therapy for adults with borderline personality disorder in a public community mental health setting. Borderline Pers Disorder Emot Dysregul.

[19] Gratz KL, Roemer L (2004). Multidimensional assessment of emotion regulation and dysregulation: development, factor structure, and initial validation of the difficulties in emotion regulation scale. J Psychopathol Behav Assess.

[20] Bohlmeijer E, ten Klooster PM, Fledderus M, Veehof M, Baer R (2011). Psychometric properties of the five facet mindfulness questionnaire in depressed adults and development of a short form. Assessment..

[21] Neacsiu AD, Rizvi SL, Vitaliano PP, Lynch TR, Linehan MM (2010). The dialectical behavior therapy ways of coping checklist: development and psychometric properties. J Clin Psychol.

[22] Braun V, Clarke V (2006). Using thematic analysis in psychology. Qual Res Psychol.

[23] Neacsiu AD, Rizvi SL, Linehan MM (2010). Dialectical behavior therapy skills use as a mediator and outcome of treatment for borderline personality disorder. Behav Res Ther.

[24] Alexander CN, Robinson P, Rainforth M (1994). Treating and preventing alcohol, nicotine, and drug abuse through transcendental meditation: a review and statistical meta-analysis. Alcohol Treat Quart.

[25] Haaga DAF, Grosswald S, Gaylord-King C, Rainforth M, Tanner M, Travis F (2011). Effects of the transcendental meditation program on substance use among university students. Cardiol Res Pract..

[26] Creswell J (2015). A concise introduction to mixed methods research.

